# Influence of two breakfast meals differing in glycemic load on satiety, hunger, and energy intake in preschool children

**DOI:** 10.1186/1475-2891-9-53

**Published:** 2010-11-12

**Authors:** Alison LaCombe, Vijay Ganji

**Affiliations:** 1 Food and Nutrition, Mayo Clinic, Scottsdale, AZ 85260, USA; 2 Division of Nutrition, School of Health Professions, College of Health and Human Sciences, Georgia State University, Atlanta, GA 30303, USA

## Abstract

**Background:**

Glycemic load (GL) is the product of glycemic index of a food and amount of available carbohydrate in that food divided by 100. GL represents quality and quantity of dietary carbohydrate. Little is known about the role of GL in hunger, satiety, and food intake in preschool children. The aim of this study was to investigate the effect of two breakfast meals differing in GL on hunger, satiety, and subsequent food intake at lunch in preschool children aged 4-6 y.

**Methods:**

Twenty three subjects consumed low-GL (LGL) and high-GL (HGL) breakfast meals according to a randomized crossover design followed by an *ad libitum *lunch 4 h after consumption of breakfast. Children were asked to consume meals until they are full. Each treatment was repeated twice in non-consecutive days and data were averaged.

**Results:**

Children in LGL group consumed significantly lower amounts of GL, total carbohydrate, energy, energy density, and dietary fiber and higher amounts of protein and fat at the breakfast compared to those in HGL group. Prior to lunch, children were hungrier in the HGL intervention group compared to the LGL intervention group (*P *< 0.03). However, no significant difference was observed between LGL and HGL intervention groups in the amount of food and energy consumed during lunch.

**Conclusions:**

Decreased hunger in children prior to lunch in LGL group is likely due to higher protein and fat content of LGL breakfast. Diets that are low in GL can be recommended as part of healthy diet for preschool children.

## Introduction

GI is defined as the incremental area under the glucose response curve after consumption of 50 g of available carbohydrate from a test food, divided by the area under the curve after consumption 50 g of carbohydrate from a reference food, glucose or white bread [1]. Thus, GI represents the quality of carbohydrate contained in foods [2]. Refined carbohydrates and sweetened foods and beverages have high glycemic index (GI), whereas foods containing complex carbohydrates, vegetables, and legumes have low GI [3]. Foods that are high in protein and fat such as meat, fish, poultry, cheese, and eggs were given no GI values because these foods contain little or no carbohydrate and hence unlikely that these foods elicit glycemic response even consumed in large quantities [3]. The GI of an average American diet has increased because of the increase in carbohydrate consumption along with changes made in the processing of foods [4-6]. This shift in carbohydrate consumption coincided with increased prevalence of overweight and obesity in most Western countries [7]. In the US >60% of adults are either overweight or obese [8]. Also, in children the prevalence of overweight and obesity continues to increase [9,10]. The prevalences of overweight and obesity in the US pre-school children are 12% and 14%, respectively [10]. Obesity is linked to the onset of several chronic diseases, such as type 2 diabetes, hypertension, and coronary heart disease (CHD) [11].

Research has shown an association between consumption of diets high in GI and obesity risk factors, type-2 diabetes, increased hunger, and increased energy consumption [12-17]. Thus, diets low in GI have a role in the management of diabetes because of improved postprandial glucose control [18,19]. Also diets low in GI compared to reduced fat diets, have been associated with greater decreases in body mass index (BMI), body weight, and fat mass in children [20]. However, the exact mechanism through which GI regulates body weight is not clearly understood.

Glycemic load (GL) is the product of GI of a food and amount of available carbohydrate present in that food divided by 100. Thus, GL represents both quality and quantity of carbohydrate containing foods [21]. Diets low in GL have been shown to be associated with a lower risk of CHD and diabetes and may be beneficial in the management of these diseases [22-26]. Very limited research has been conducted on the relation between GL and body weight, satiety, hunger and food intake [27,28]. Ebelling et al [27] reported that a reduced GL diet has resulted in a greater decrease in fat mass and a lesser increase in insulin resistance in comparison to a conventional low-fat diet. The effect of GL on satiety, hunger, or energy intake in preschool children has never been studied. The aim of this study was to investigate the effect of two test breakfast meals differing in GL, low-GL (LGL) and high-GL (HGL), on satiety, hunger, and energy intake in the subsequent meal in preschool children.

## Methodology

### Subjects and study design

The study commenced after obtaining the approval by the Institutional Review Board at Rush University Medical Center (RUMC), Chicago. A written consent was obtained from the legal guardians of all the children who participated in the study. Preschool children aged 4-6 years old from the RUMC's day school were recruited for this study. From a pool of children enrolled in the day school, 25 children participated in the study. Inclusion criteria for participation were that the child be in good health, regularly attend the day care facility, and regularly participate in the breakfast meal provided by the day care facility. Children with diabetes and heart disease, or who had food allergies, diet restrictions, or any other diet-related conditions that would prevent them from eating certain foods included in the test meals were excluded from participation. Of 25 children recruited, 2 children dropped out of the study due to unknown reasons. Twenty three subjects completed the study. Study sample consisted of 16 boys and 7 girls of diverse ethnic backgrounds. Characteristics of study population are presented in Table 1.

**Table 1:** Demographic characteristics of preschool children^1^

Characteristic	Total	Boys	Girls	***P*-value**^**2**^
	(n = 23)	(n = 16)	(n = 7)	
Race-ethnicity				
Non-Hispanic white	6	5	1	-
Non-Hispanic black	8	6	2	-
Mexican American/Hispanic	3	3	0	-
Others	6	2	4	-
Age, *y*	4.6 ± 0.7	4.8 ± 0.7	4.1 ± 0.4	0.033
Weight, *kg*	21.1 ± 3.3	21.2 ± 3.5	21.0 ± 3.2	0.922
Height, *cm*	114 ± 5.9	115 ± 6.3	113 ± 5.2	0.820
Body mass index^3^	16.1 ± 1.3	16.0 ± 1.3	16.3 ± 1.4	0.769

^1 ^Values are mean ± standard deviation

^2 ^Comparison between boys and girls. Significance in the Mann-Whitney U test. Data were non-normal

^3 ^Bodyweight in kg/height in m^2^

The dietary intervention was according to a randomized crossover design and consisted of 4 separate intervention days for each subject. Participants were randomly assigned to two breakfast interventions, LGL and HGL. In week A of the intervention, 11 subjects received the LGL test breakfast meal and 12 subjects received the HGL test breakfast meal. In week B, the subjects were crossed over to the alternate test breakfast meal. In both weeks, intervention was performed on two non-consecutive days. There was a 3 days of gap before the intervention was repeated. This served as a washout period between duplicate interventions. The data from these two days were averaged to yield a reliable estimate. Menus were identical on both days of intervention. Children consumed both test breakfast meals and lunch meals in the classroom to ensure a normal meal setting and to reduce the likelihood of confounding by change in environment.

Children were asked to abstain from consumption of food and beverage other than water prior to their arrival in the morning on the day of study intervention. Menu composition of test breakfast meals differing in GL is presented in Table 2. Food items for breakfast and lunch were portioned, weighed, and labeled appropriately for each subject. Breakfast was served between 8:00 AM and 9:00 AM and lunch was served 4 h after the breakfast meal concluded. Lunch consisted of an *ad libitum *meal that was selected from the facility's lunch menu. Lunch menu consisted of cheese ravioli or macaroni and cheese, green beans or mustard greens, French bread or corn bread, mixed fruit cup or pudding, margarine, and whole milk. Children were asked to consume breakfast and lunch meals until they are full. All breakfast and lunch food items were weighed prior to consumption and all leftover food (plate-waste) was weighed after breakfast and lunch.

**Table 2:** Menu composition of two test breakfast meals differing in GL served to preschool children^1^

Food	Quantity of food (*g*)	GI	Quantity of carbohydrate (*g*)	**GL**^**2**^
HGL breakfast				
Cornflakes	34	92	29.6	27.2
Whole milk	170	40	7.7	3.1
Banana	74	51	16.9	8.6
LGL breakfast				
Egg substitute	50	0	0.5	0
Strawberries	46	40	3.5	1.4
Whole milk	170	40	7.7	3.1

^1 ^Abbreviations: GI, glycemic index; GL, glycemic load; HGL, high-glycemic load; LGL, low-glycemic load

^2 ^GL, calculated as [GI × carbohydrate (g)]/100. Carbohydrate content was determined using the USDA nutrient data base

### Measurements

The nutrient composition of test breakfast meals are presented in Table 3. Nutrient analysis of foods was calculated using the USDA National Nutrient Database [29]. The GL value for each food was determined using the GI values published in the International Tables of Glycemic Index and Load and multiplied by the amount of available carbohydrate eaten by the subject. Then the GL value for each meal was calculated by summing the GL values for each food consumed. The amount of food consumed at breakfast and lunch was calculated by the difference in weight of food served to the subjects in comparison to the weight of food remaining after consumption (plate-waste).

**Table 3:** Nutrient Composition of two test breakfast meals differing in GL served to preschool children^1^

**Nutrient**^**2**^	LGL	HGL
GL^3^	4.5	38.9
Quantity of food, *g*	266	278
Energy content, *kcal*	175.5	290.6
Carbohydrate, g (%)	11.7 (27)	54.2 (75)
Protein, g (%)	14.2 (32)	8.5 (12)
Fat, g (%)	8.0 (41)	6.0 (18)
Fiber, g (g/1000 kcal)	0.9 (5.1)	2.8 (9.6)

^1^Abbreviations: GL, glycemic load; HGL, high-glycemic load; LGL, low-glycemic load

^2^According to the USDA nutrient database

^3^GL, calculated as [GI × carbohydrate (g)]/100

Hunger prior to breakfast and lunch, palatability of breakfast and lunch meals, and satiety after breakfast and lunch were measured in both treatments. A 5-point scale was used in measurement of hunger before breakfast and lunch (1 = very empty, 3 = empty, 5 = a little empty, 7 = normal-not empty/full, 9 = not empty at all), satiety after breakfast and lunch (1 = not full at all, 3 = normal-not full/empty, 5 = a little full, 7 = full, 9 = very full), and palatability of breakfast and lunch meals (1 = very bad, 3 = bad, 5 = okay, 7 = good, 9 = very good). On the scale, each number had a corresponding pictorial smiley face. Each subject was interviewed individually using a preprepared script by the same researcher to collect the data on palatability of breakfast and lunch meals, hunger before breakfast and lunch meals, and satiety after breakfast and lunch meals. Each interview with children took 2-3 minutes. BMI was calculated from weight and height measurements (weight in kg/height in m^2^)

### Statistical Analysis

Data were analyzed using Microsoft Excel 2003 and SPSS version 11.5 (SPSS, Inc., Chicago, IL). Differences in characteristics of the study population between boys and girls were performed with Mann Whitney U test (non-normal data). Differences between LGL and HGL groups in energy intake, amount of food eaten, nutrient density, hunger, satiety, and palatability ratings for breakfast and lunch meals were analyzed with the Wilcoxon Signed Rank Test (non-normal data). A t-test was performed to assess if a significant difference existed between GL intake values of LGL and HGL test breakfast meals (normal data). Statistical significance was set at an *α *value of 0.05.

## Results

No significant differences were found between boys and girls for weight (*P *= 0.922), height (*P *= 0.820), or BMI (*P *= 0.769). Boys were significantly older than girls (*P *= 0.033). One girl was considered obese and 4 boys were considered overweight [30].

Intakes of GL and nutrients consumed by the children for each breakfast group are presented in Table 4. GL intakes for the LGL and HGL test breakfast groups ranged from 1.1 to 4.0 and 9.0 to 37.4, respectively. As expected, a significant difference was observed between the GL intakes of LGL and HGL groups (*P *< 0.0001). Intakes of total energy, energy density, carbohydrate, and dietary fiber were significantly lower, while intakes of protein and fat were significantly higher in the LGL group compared to the HGL group. No significant difference was observed in the amount of food consumed between the LGL and the HGL intervention groups during breakfast. Also, no significant differences were observed between LGL and HGL groups in intakes of total quantity of food, total energy, and energy density during lunch.

**Table 4:** Food and nutrient intakes by preschool children consuming two test breakfast meals differing in GL and lunch meals^1, 2^

**Macronutrient**^**3**^	LGL	HGL	***P*-value**^**4**^
Breakfast			
GL^5^	2.5 ± 0.9	23.1 ± 8.6	<0.001
Quantity of food, *g*	154 ± 54	150 ± 51	NS
Energy, *kcal*	104 ± 39	165 ± 55	<0.001
Energy density, *kcal/g*	0.7 ± 0.1	1.1 ± 0.2	0.001
Carbohydrate, g (%)	6.6 ± 2.2 (25)	32 ± 11 (78)	<0.001
Protein, g (%)	8.8 ± 3.7 (34)	4.6 ± 1.8 (11)	0.001
Fat, g (%)	4.6 ± 2.0 (40)	3.0 ± 1.4 (16)	0.003
Fiber, g (g/1000 kcal)	0.6 ± 0.4 (5.8)	1.7 ± 0.8 (10.3)	<0.001
Lunch			
Quantity of food, *g*	349 ± 113	359 ± 106	NS
Energy, *kcal*	404 ± 171	395 ± 123	NS
Energy density, *kcal/g*	1.1 ± 0.2	1.1 ± 0.2	NS

^1^Abbreviations: Abbreviations: GL, glycemic load; HGL, high-glycemic load; LGL, low-glycemic load; NS, non-significant (*P *> 0.05)

^2^Values are mean ± standard deviation and percentages in parentheses

^3^According to the USDA nutrient database

^4^Comparison between LGL and HGL test breakfasts. Wilcoxon Signed Ranks Test. t-test was used only for GL because of normality if the data.

^5^GL, calculated as [GI × carbohydrate (g)]/100

A significant difference was found between hunger scores before lunch in children 4 h after consumption of breakfast meals differing in GL (*P *= 0.03) indicating children were hungrier in the HGL intervention group compared to the LGL intervention group (Table 5). No significant difference was observed between palatability scores of LGL and HGL meals. Satiety scores after breakfast and lunch and hunger scores before breakfast were also not significantly different between LGL and HGL interventions groups (Table 5).

**Table 5:** Hunger, palatability, and satiety scores for test breakfast meals differing in GL and for lunch meals as consumed by preschool children^1, ^^2^

Outcome	LGL	HGL	***P*-value**^**3**^
	(n = 23)	(n = 23)	
Hunger before breakfast^4^	2.3 ± 1.4	1.9 ± 1.1	NS
Palatability of breakfast^5^	7.9 ± 1.5	8.4 ± 1.0	NS
Satiety after breakfast^6^	7.1 ± 3.5	7.2 ± 3.6	NS
Hunger before lunch^4^	2.5 ± 1.7	1.8 ± 1.1	0.03
Palatability of lunch^5^	8.5 ± 0.8	8.4 ± 1.1	NS
Satiety after lunch^6^	7.8 ± 1.3	7.9 ± 1.4	NS

^1^Abbreviations: HGL, high-glycemic load; LGL, low-glycemic load; NS, non-significant (*P *> 0.05)

^2^Values are mean ± standard deviation

^3^Comparison between LGL and HGL test breakfast meals, Wilcoxon Signed Ranks Test

^4^Hunger Scale: 1 = "very empty", 3 = "empty", 5 = "a little empty", 7 = "normal-not empty/full", 9 = "not empty all"

^5^Palatability Scale: 1 = "very bad", 3 = "bad", 5 = "okay", 7 = "good", 9 = "very good"

^6^Satiety Scale: 1 = "not full at all", 3 = "normal-not full/empty", 5 = "a little full", 7 = "full", 9 = "very full

## Discussion

In this study, we investigated the effect of 2 breakfast meals differing in GL on satiety after breakfast, hunger prior to lunch, and energy intake at lunch in pre-school children aged 4-6 y old. We observed that children were less hungry in the LGL intervention compared to the HGL intervention group. However, this reduced hunger prior to lunch in the LGL intervention group did not result in a significant reduction either in energy intake or in the quantity of food consumed at lunch. Also, there was no significant difference in satiety ratings after breakfast between the LGL and HGL intervention groups. Additional analysis revealed no correlation between pre-lunch hunger scores and subsequent energy intake for either of the test breakfast meals (data not shown), suggesting that the pre-lunch hunger had no significant effect on lunch energy intakes in preschool children.

In contrast, recently, Fajcsak et al [31] in Hungarian pre-pubertal overweight/obese children aged 11 y found that the self-reported hunger was significantly reduced after consumption of diets high in GL. A few other studies have reported on the effect of GI on food intake and hunger. Ludwig et al [13] found that obese teenagers not only had an increase in hunger before lunch following consumption of the high-GI breakfast, but that they also consumed significantly more energy at lunch following the high-GI breakfast in comparison to the medium GI breakfast (*P *< 0.05) and the low-GI breakfast (*P *= 0.01). Warren et al [14] found that after consumption of the high-GI breakfast meal, energy intake at lunch was 145 kcal higher than the low-GI breakfast meal and 119 kcal higher than the low-GI plus sucrose breakfast meal (*P *≤ 0.05). In contrast, Ball et al [15] found no significant difference in energy intake at the subsequent meal between those on the high-GI meal replacement and those on the low-GI meal replacement or the low-GI whole foods meal. Alfenas and Mattes [32] found no significant difference in food intake between persons consuming low and high GI foods. These observations are in line with our findings, although we studied the impact of GL rather than GI on food intake in a subsequent meal.

The increased hunger that is experienced with the HGL meal may be related to the hormonal and metabolic consequences of HGL foods. Consumption of a HGL meal leads to rapid absorption of glucose because HGL foods are more readily digestible [33]. The counter regulatory hormone, glucagon is inhibited by elevated glucose and gut hormones, while release of insulin is stimulated [33]. The high insulin concentration promotes glucose uptake by liver and muscle, while suppressing lipolysis in adipoctyes and reducing the release of glucose from the liver into the circulation [13,34]. As a result, the blood glucose concentration is rapidly decreased following a HGL meal when compared to a LGL meal [33]. Thus, the hunger response occurs faster with a HGL meal than with a LGL meal [13]. This increased hunger may or may not lead to increased energy intake in subsequent meal. In the present study, greater energy intake at lunch after consuming the HGL test breakfast meal did not occur indicating that the association between hunger and energy intake is much more complicated than a simple linear association.

Additional analysis revealed no correlation between intake of breakfast GL value and satiety after breakfast, hunger prior to lunch, or energy intake at lunchtime during either of the test breakfast interventions. This led us to believe that the significantly greater hunger we observed before lunch after consumption of HGL breakfast was due to factor(s) other than the GL of the breakfast meal consumed. Although attempts were made to match macronutrient contents of the two test breakfast meals, children were allowed to consume as they desired, therefore macronutrient intake varied from child to child. In the LGL intervention group, children consumed significantly more protein and fat compared to HGL group at the breakfast. Protein and fat are known to trigger the release of cholecystokinin (CCK) from I cells of the duodenal and jejunal mucosal cells. CCK activates CCK receptor-1 in the pyloric sphincter leading to pyloric sphincter contraction and decreased gastric emptying [35]. This further leads to decreased hunger. Dietary fiber could not have played a role in decreased hunger in the LGL intervention group because the dietary fiber intake was significantly lower in the LGL than in the HGL intervention group. Therefore, the significant difference observed between the test breakfast meals in hunger before lunch may be due to significant differences in the macronutrient intakes associated with two test breakfast meals.

Despite the lack of significant difference observed in hunger before breakfast for the two test breakfast meals, significantly more energy was consumed by children at breakfast when the HGL test breakfast was served. However, no significant difference was found in the amount of food consumed at breakfast between the LGL and HGL intervention groups. This can be attributed to the greater energy density of the HGL test breakfast meal than the LGL test breakfast meal. The energy density of the food consumed by the HGL intervention group was ≈0.4 kcal/g higher than the energy density of the food consumed by the LGL group. Foods with high GL tend to have a greater energy density due to the fact that they are usually processed as convenience-type foods and also often have greater sugar contents [3,36].

In conclusion, this study suggests that when pre-school aged children consumed breakfast meals with differing GLs, a significant difference in hunger before lunch resulted. However, the observed difference in hunger prior to lunch did not have an impact on energy intake at lunch. It is possible that the significant difference observed in huger prior to lunch was due to difference in micronutrient intakes from these test meals. One limitation of this study was that the children regularly chose extreme ratings due to their inability to fully understand the meaning of hunger, satiety, or palatability. Another limitation of this study is that the results may have been confounded by the energy content, carbohydrate quantity and quality, fiber content, and glycemic index of breakfast meals. In this study, participants were not required to consume the entire portion of the breakfast. However, it is not known how this affected the study outcomes. Studies are needed to validate hunger, satiety, and palatability scales in pre-school age children. More research is needed to establish a clear role of GL in hunger and satiety, and its eventual relation with obesity in various stages of life.

## Competing interests

The authors declare that they have no competing interests.

## Authors' contributions

AL and VG designed the study, collected the data, analyzed the data, and wrote the manuscript. Both authors edited and approved the final manuscript.
